# Birth outcome disparities and immigrant paradox among Southeast Asian migrant and Thai mothers during the COVID-19 pandemic: a retrospective cohort study

**DOI:** 10.25122/jml-2025-0128

**Published:** 2025-12

**Authors:** Somruethai Khamsakhon, Chanapong Rojanaworarit, Isabella Andrade, Worawaran Kallayanasit, Panunda Yodkhunnathum, Thunyaporn Sirijantradilok, Supasit Suerungruang, Nuttawoot Photisan

**Affiliations:** 1 Department of Disease Control, Institute of Preventive Medicine, Ministry of Public Health, Nonthaburi, Thailand; 2 Department of Population Health, School of Health Sciences, Hofstra University, Hempstead, NY, USA; 3 Master of Public Health Program, Hofstra University, Hempstead, NY, USA; 4 Somdejphrajaotaksin Maharaj Hospital, Tak, Thailand; 5 Health Strategic Development Group, Trat Provincial Public Health Office, Trat, Thailand

**Keywords:** preterm birth, low birth weight, migrant, COVID-19, epidemiology

## Abstract

This study aimed to evaluate disparities in preterm birth and low birth weight among newborns of mainland Southeast Asian migrant versus Thai mothers during the COVID-19 pandemic in Thailand, and to assess the immigrant paradox by examining associations between maternal migrant status and adverse birth outcomes using directed acyclic graphs. The study population consisted of mainland Southeast Asian migrant and Thai mothers and their newborns who received antenatal care and gave birth at a public hospital in Samut Sakhon from December 2020 to May 2022. Preterm birth (gestational age < 37 weeks) and low birth weight (< 2500 g) were the study outcomes. Migrant status was defined using personal identification (e.g., passport). Associations between migrant status and birth outcomes, adjusted for pertinent covariates, were modeled using a directed acyclic graph. Poisson regression with robust standard errors was applied to estimate the risk ratio. Preterm birth incidence was 10% and 9% among newborns of migrant and Thai mothers. Low birth weight incidence was significantly higher among newborns of Thai mothers (10.7%) compared to those of migrant mothers (8%). After adjusting for covariates, immigrant status did not increase the risk of preterm birth or low birth weight in newborns compared to those of Thai mothers. Contrary to the assumption that migrant mothers may face hardships leading to newborns’ poor birth outcomes, risk of preterm birth and low birth weight were similar among newborns of migrant and local mothers in this study, indicating an immigrant health paradox.

## INTRODUCTION

Comparisons of health outcomes between local and migrant populations are instrumental in explaining disparities and identifying the healthcare needs of migrants. Evidence indicates that both documented and undocumented migrant mothers frequently encounter barriers to adequate prenatal, natal, and postnatal care, such as language obstacles, limited engagement in medical decision-making, and cultural differences, which may adversely affect maternal and neonatal outcomes [1-8]. Interventions that have aimed to target disparities in birth outcomes, such as low birth weight and preterm birth, have seen success in recruiting individuals in preventive care with the utilization of linguistically and culturally appropriate design [8-10]. However, previous studies have identified the ‘immigrant paradox’, in which migrants appear to have better health outcomes than their native-born counterparts. This paradox may be attributable to migrant cultural behaviors that contribute to these better health outcomes, and evidence suggests that this paradox disappears as migrants begin to acculturate in the host country; however, the intersectionality of multiple factors, rather than migrant status alone, may be responsible for disparities between native-born populations and migrants [11-14].

Studies previously examining the disparities in birth outcomes between the newborns of migrant and native mothers were mainly conducted in Western countries [5,6,13,14]. Nonetheless, comparable investigations with similar scope and depth remain limited in mainland Southeast Asia. In Thailand, a previous study in 2011 reported the incidence of low birth weight to be higher among the newborns of migrant mothers compared to those of native mothers, while the incidence of preterm birth appeared comparable between the two groups [15]. This evidence, however, predates the unprecedented impact of the recent COVID-19 pandemic, which has been hypothesized to widen disparities in birth outcomes. Considering the effects of SARS-CoV-2 infection on migrant populations [16,17] and pregnant women [18,19], the COVID-19 pandemic has provided a unique opportunity to further examine disparities in birth outcomes by migrant status and SARS-CoV-2 infection status. This study attempted to evaluate the birth outcomes potentially affected by the COVID-19 pandemic and its associated challenges, within a context that has received limited prior investigation. Additionally, despite the multitude of factors that intersect to create disparities in birth outcomes, the application of directed acyclic graphs (DAGs) in study design and data analysis has been rare in birth outcome studies. Using the DAG models, a causal inference framework, would not only validate the prior findings but also advance methodological rigor and provide insight into the interplay of social and clinical determinants of adverse birth outcomes.

The primary objective of the study was to assess disparities in preterm birth and low birth weight by comparing their incidence among newborns of mainland Southeast Asian migrant and Thai mothers during the peak COVID-19 pandemic in Samut Sakhon, Thailand. This study also aimed to evaluate the immigrant paradox by assessing the association between maternal migrant status, a social determinant of interest, and the clinical outcomes of preterm birth and low birth weight, sequentially adjusted for covariates according to epidemiological models suggested by DAGs.

## MATERIAL AND METHODS

### Study design and setting

This retrospective cohort study analyzed clinical data from newborns and their mothers collected during antenatal (ANC), natal, and postnatal care services at a public hospital in Samut Sakhon Province, Thailand, between December 3, 2020, and May 3, 2022. Samut Sakhon, a coastal province on the Gulf of Thailand, is home to a large seafood industry and to one of the country’s biggest concentrations of foreign migrant workers from neighboring mainland Southeast Asian countries, such as Myanmar and Cambodia, as well as from non-Thai ethnic minority groups, including hill tribes. This study context was chosen to investigate health inequities among documented and undocumented foreign migrants stemming from employment in low-wage, labor-intensive jobs within the fishery industry, poor living conditions, language barriers, psychological stress from migrant status, and barriers to healthcare access that may have been exacerbated during the COVID-19 pandemic. This province was the epicenter of the December 17, 2020, COVID-19 outbreak that triggered the second wave across Thailand, providing a unique opportunity to examine the impact of SARS-CoV-2 infection on health outcomes.

### Study population

Mothers and their newborns who attended ANC, were tested for SARS-CoV-2 infection by real-time reverse transcription polymerase chain reaction (RT-PCR) or a rapid diagnostic test (RDT), and delivered at the study facility between December 3, 2020, and May 3, 2022, were eligible for inclusion in the cohort. Maternal data were matched to corresponding newborn records. Exclusion criteria included mothers who received abortion care, experienced stillbirth, or had a gestational age (GA) of ≥14 weeks at or within two weeks before the onset of the local COVID-19 outbreak on December 17, 2020. In total, 31 abortion cases and two stillbirths were excluded from the study.

A minimal sample size of 520 newborns was estimated for this study, assuming an alpha error of 0.05 and a statistical power of 80%. The assumed ratio of foreign migrants to Thai mothers was 1:1 based on records in the ANC at this facility. Proportions of low-birth-weight newborns born to foreign migrant and Thai mothers were 22% and 12.7% [15]. For the regression analysis, the required sample size was further determined by considering the effect size and the number of predictor variables. Assuming a medium effect size of 0.15 and including eight predictor variables, the required minimum sample size was 108 based on an a priori power analysis. Ultimately, 1,971 newborns were included for analysis.

### Outcome measurement

Two adverse birth outcomes of interest were preterm birth and low birth weight. To identify preterm birth, GA at birth was initially calculated using the date of birth minus the date of the last menstrual period (LMP). GA < 37 weeks was defined as a preterm birth. Low birth weight was defined as having a birth weight < 2,500 grams [20]. Data regarding GA, birth weight, and natal sex were obtained from perinatal care records in the hospital information system (HIS).

### General and obstetric characteristics of the participants

The primary independent variable was maternal migrant status, categorized as foreign migrant or Thai national. Foreign migrants included individuals with a foreign passport, non-Thai identification card, or work permit (documented), as well as those lacking such documents (undocumented). Thai nationals were identified by a Thai identification card. Maternal characteristics included age, healthcare coverage, preexisting medical conditions (e.g., diabetes mellitus), pregnancy complications (e.g., premature rupture of membranes [PROM]), SARS-CoV-2 infection during pregnancy, and receipt of the COVID-19 vaccine before delivery. Data regarding maternal age and healthcare coverage were obtained from records at the first ANC visit. Details of different benefit packages for healthcare coverage are provided in the Supplementary File 1. Medical conditions and complications during pregnancy were diagnosed according to the International Classification of Diseases 10^th^ Revision (ICD-10). HIV, syphilis, SARS-CoV-2 infection, and pregnancy anemia were confirmed by laboratory tests. All these variables were retrieved from HIS.

### Data collection

Data were extracted from the HIS, comprising routinely recorded service-based information from antenatal care, delivery, and neonatal services. As secondary data were utilized, active participant recruitment was not required. Access to electronic health records was granted by the hospital director after ethical approval. Data confidentiality was maintained by de-identifying all records. The data were stored on an encrypted computer, with access restricted to authorized researchers.

**Figure 1:**
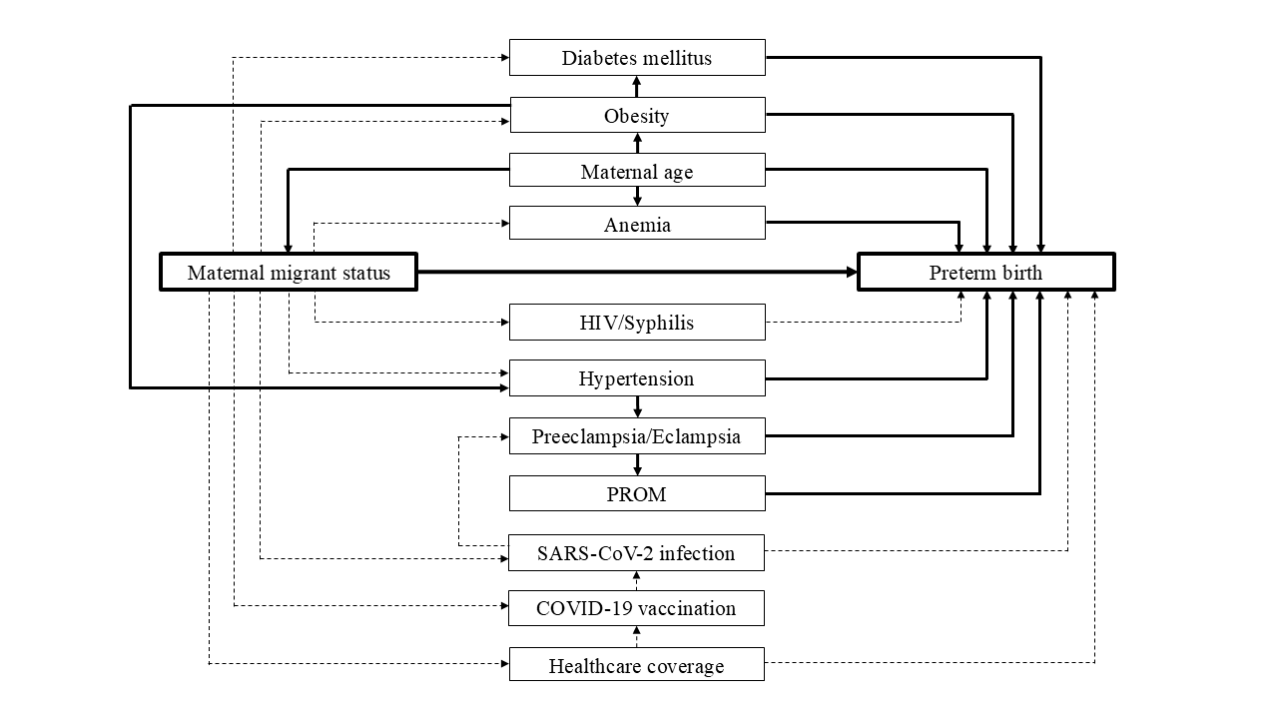
Statistical models according to directed acyclic graphs for preterm birth outcome. Model 1. Crude model of maternal migrant status (main independent variable or exposure, E) and preterm birth (dependent variable, D): E → D. Model 2. Model of (E) and (D) adjusted for maternal age (A): E ← A → D. Model 3. Model of (E) and (D) adjusted for maternal age (A) and obesity (O): E ← A → O → D. Model 4. Model of (E) and (D) adjusted for maternal age (A), obesity (O), and diabetes mellitus (M): E ← A → O → M → D. Model 5. Model of (E) and (D) adjusted for maternal age (A), obesity (O), and hypertension (H): E ← A → O → H → D. Model 6. Model of (E) and (D) adjusted for maternal age (A), obesity (O), hypertension (H), and preeclampsia/eclampsia (P): E ← A → O → H → P → D. Model 7. Model of (E) and (D) adjusted for maternal age (A), obesity (O), hypertension (H), preeclampsia/eclampsia (P), and premature rupture of membranes (R): E ← A → O → H → P → R → D. Model 8. Model of (E) and (D) adjusted for maternal age (A) and anemia (N): E ← A → N → D. *COVID-19, coronavirus disease; HIV, human immunodeficiency virus; PROM, premature rupture of membranes; SARS-CoV-2, severe acute respiratory syndrome coronavirus 2 **Dashed lines denote causal paths; solid lines denote non-causal paths.

### Statistical analysis

The characteristics of participants were summarized using descriptive statistics. To obtain the crude and adjusted estimates of the association between maternal migrant status and the risk of preterm birth and low birth weight, Poisson regression with robust standard errors was applied. The two binary outcomes were preterm birth (index group: GA < 37 weeks, referent group: GA ≥ 37 weeks) and low birth weight (index group: birth weight < 2500 grams, referent group: birth weight ≥ 2500 grams). The multivariable regression analyses were sequentially executed to adjust for different sets of potential confounders according to epidemiological causal models illustrated by DAGs for each outcome.

**Figure 2:**
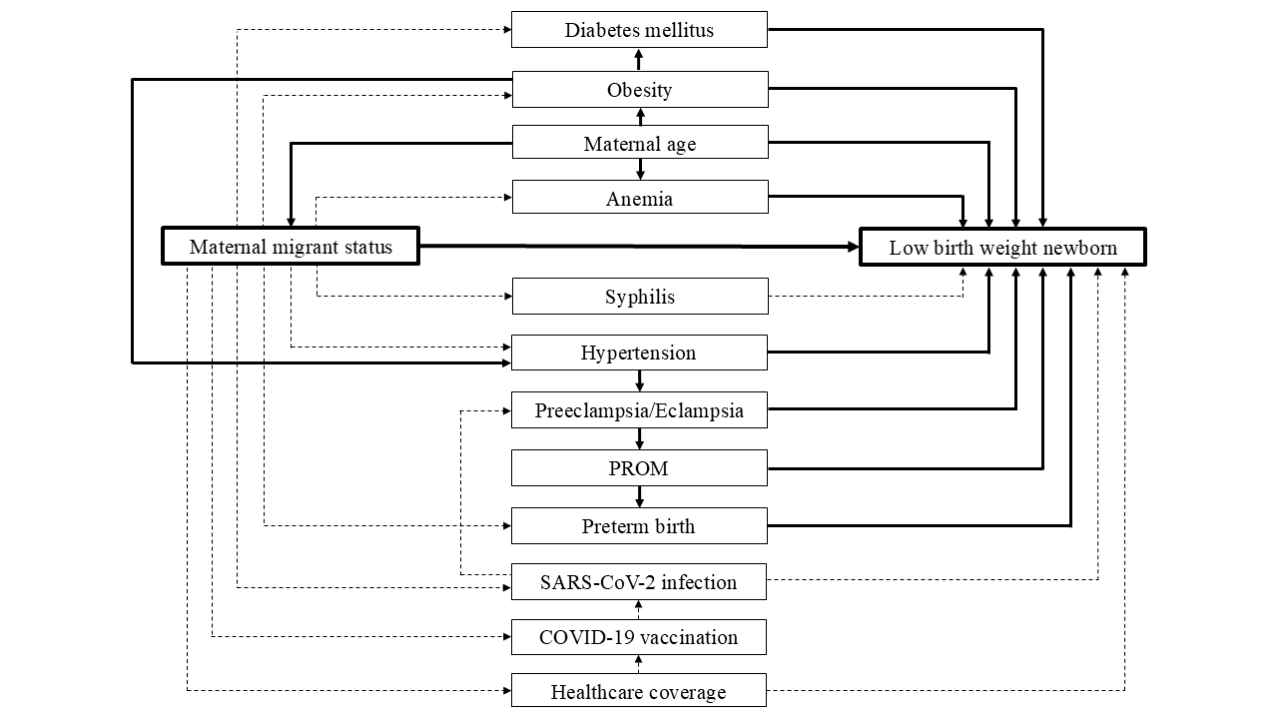
Statistical models according to directed acyclic graphs for low-birth-weight outcome. Model 1. Crude model of maternal migrant status (main independent variable or exposure, E) and low birth weight (dependent variable, D): E → D. Model 2. Model of (E) and (D) adjusted for maternal age (A): E ← A → D. Model 3. Model of (E) and (D) adjusted for maternal age (A) and obesity (O): E ← A → O → D. Model 4. Model of (E) and (D) adjusted for maternal age (A), obesity (O), and diabetes mellitus (M): E ← A → O → M → D. Model 5. Model of (E) and (D) adjusted for maternal age (A), obesity (O), and hypertension (H): E ← A → O → H → D. Model 6. Model of (E) and (D) adjusted for maternal age (A), obesity (O), hypertension (H), and preeclampsia/eclampsia (P): E ← A → O → H → P → D. Model 7. Model of (E) and (D) adjusted for maternal age (A), obesity (O), hypertension (H), preeclampsia/eclampsia (P), and premature rupture of membranes (R): E ← A → O → H → P → R → D. Model 8. Model of (E) and (D) adjusted for maternal age (A) and anemia (N): E ← A → N → D. *COVID-19, coronavirus disease; PROM, premature rupture of membranes; SARS-CoV-2, severe acute respiratory syndrome coronavirus 2 **Dashed lines denote causal paths; solid lines denote non-causal paths.

Paths of DAGs for preterm birth and low birth weight outcomes were depicted in Figure 1 and Figure 2, respectively. According to the DAGs, the outcome or dependent variable in each statistical model was denoted as ‘D.’ Model 1 was a crude (unadjusted) model that included only the main independent variable, maternal migrant status (E). Model 2 included (E) and adjusted for maternal age (A). Regressors in Model 3 comprised (E), (A), and obesity (O). Regressors in Model 4 were (E), (A), (O), and diabetes mellitus (M). Regressors in Model 5 included (E), (A), (O), and hypertension (H). Preeclampsia/eclampsia (P) was adjusted along with (E), (A), (O), and (H) in Model 6. Regressors in Model 7 included those in Model 6 but additionally adjusted for premature rupture of membranes (R). Regressors in Model 8 included (A) and anemia (N).

## RESULTS

### General and obstetric characteristics of mothers and neonatal characteristics

Slightly over half (51.3%) of all 1,957 mothers were foreign migrant mothers. The foreign migrant mothers were mostly Burmese (87%). The average age of Thai mothers was significantly lower than that of the migrant mothers. Around 17.9% of Thai mothers were teenagers (age < 20), whereas only 1.9% of foreign migrant mothers were teenagers. A much greater proportion of mothers in the optimal reproductive age (20 ≤ age < 35) was found among foreign migrant mothers (80.3%) compared to that of Thai mothers (68.3%). Migrant mothers had a significantly lower proportion of coverage under health insurance (41.7%) than Thai mothers (91.6%). Among Thai mothers with healthcare coverage, the share of healthcare coverage schemes was 47% for the Universal Healthcare Coverage Scheme (UCS), 42.9% for the Social Security Scheme (SSS), and 1.7% for the Civil Servant Medical Benefit Scheme (CSMBS). In contrast, more than half of migrant mothers were uninsured and needed to pay out of pocket for health services. The uninsured subgroups of migrant mothers were the documented migrant mothers without coverage by the Health Insurance Care Scheme (HICS) or SSS (55.2%) and the undocumented migrant mothers (3%; Table 1). Details of health care coverage schemes for Thai and migrant mothers were further explained in the Supplementary File 1.

Analysis of maternal clinical data revealed an overall incidence of SARS-CoV-2 infection during pregnancy of 13.3% (261 cases out of all mothers). A significantly higher incidence of SARS-CoV-2 infection during pregnancy was observed among foreign migrant mothers, 14.8% or 149 cases from 1004 migrant mothers, compared to 11.8% or 112 cases from 953 Thai mothers, according to the exact probability test (*P* = 0.046). Regarding medical conditions, a significantly higher proportion of syphilis during pregnancy was found among Thai mothers (5%) compared to migrant mothers (1.2%). In the subgroup of mothers with SARS-CoV-2 infection, the proportion of Thai mothers with syphilis (7.6%) was also significantly greater than that of migrant mothers (1.5%). Conversely, both in the total and in the subgroups of mothers with SARS-CoV-2 infection, obesity was identified in a significantly larger proportion of migrant mothers than in Thai mothers. Incidence of pregnancy complications, such as preeclampsia and PROM, was not significantly different between Thai and migrant mothers. A significantly greater percentage of Thai mothers (68%) received the COVID-19 vaccination before delivery than migrant mothers (60.7%). Distributions of Thai and migrant mothers by varying numbers of COVID-19 vaccine doses received before delivery were also significantly different, with a noticeably greater proportion of Thai mothers (4.5%) receiving a third dose of vaccine compared to 0.6% among migrant mothers (Table 1).

The average GA at birth was significantly longer among newborns of migrant mothers. While all the newborns of Thai mothers had < 42 weeks of GA at birth, four newborns of migrant mothers had ≥ 42 weeks of GA at birth, with a maximum of 44.1 weeks. The overall incidence of preterm birth was 9.5%. Nonetheless, there was no significant difference between the 10% and 9% incidence of preterm birth among newborns of Thai and migrant mothers (*P* = 0.489). The average birth weight for all newborns was 3075.6 grams. A significantly greater average birth weight was found among newborns of migrant mothers. Birth weight varied greatly, ranging from 845 to 5252 grams. The extremely low birth weight of 845 grams was a female newborn from a Thai mother with a GA of 26 weeks. The overall incidence of low birth weight (< 2500 grams) was 9.3%. The incidence of low birth weight among newborns of Thai mothers (10.7%) was significantly higher than 8% in newborns of migrant mothers (*P* = 0.044; Table 1).

**Table 1:** General and obstetric characteristics of mothers, neonatal characteristics, and adverse birth outcomes in total and stratified by maternal SARS-CoV-2 infection status

Characteristics	Infected			*P*-value	Non-infected			*P*-value	Overall			*P*-value
	Total	Migrant	Thai		Total	Migrant	Thai		Total	Migrant	Thai	
	*n* (%)^a^	*n* (%)^a^	*n* (%)^a^		*n* (%)^a^	*n* (%)^a^	*n* (%)^a^		*n* (%)^a^	*n* (%)^a^	*n* (%)^a^	
**Participants^b^**	261 (100)	149 (57.1)^b^	112 (42.9)^b^		1,696 (100)	855 (50.4)^b^	841 (49.6)^b^		1,957 (100)	1,004 (51.3)^b^	953 (48.7)^b^	
**Maternal characteristics (*n* = 1,957)**												
Nationality												
Thai (Local)	112 (42.9)	-	112 (100)	-	841 (49.6)	-	841 (100)	-	953 (48.7)	-	953 (100)	-
Burmese	129 (49.4)	129 (86.6)	-		745 (43.9)	745 (87.1)	-		874 (44.6)	874 (87.0)	-	
Cambodian	10 (3.8)	10 (6.7)	-		51 (3.0)	51 (6.0)	-		61 (3.1)	61 (6.1)	-	
Laotian	6 (2.3)	6 (4.0)	-		53 (3.1)	53 (6.2)	-		59 (3.0)	59 (5.9)	-	
Vietnamese	1 (0.4)	1 (0.7)	-		0 (0)	0 (0)	-		1 (0.1)	1 (0.1)	-	
Shan	1 (0.4)	1 (0.7)	-		0 (0)	0 (0)	-		1 (0.1)	1 (0.1)	-	
Unspecified (non-Thais)	2 (0.8)	2 (1.3)	-		6 (0.4)	6 (0.7)	-		8 (0.4)	8 (0.8)	-	
Age at first ANC, years												
Mean ± SD	27.5 ± 6.0	28.8 ± 5.5	25.8 ± 6.3	<0.001^**^	27.7 ± 6.3	28.9 ± 5.5	26.5 ± 6.8	<0.001^*^	27.7 ± 6.3	28.9 ± 5.5	26.4 ± 6.7	<0.001^*^
Min. – Max.	14 – 43	20 – 43	14 – 43		12 – 48	14 – 48	12 – 43		12 – 48	14 – 48	12 – 43	
Teenage (<20)	21 (8.1)	0 (0)	21 (18.8)	<0.001^***^	169 (10.0)	19 (2.2)	150 (17.8)	<0.001^***^	190 (9.7)	19 (1.9)	171 (17.9)	<0.001^***^
Normal age (20–34)	202 (77.3)	122 (81.9)	80 (71.4)		1,255 (74.0)	684 (80.0)	571 (67.9)		1,457 (74.5)	806 (80.3)	651 (68.3)	
Advanced age (≥35)	38 (14.6)	27 (18.1)	11 (9.8)		272 (16.0)	152 (17.8)	120 (14.3)		310 (15.8)	179 (17.8)	131 (13.8)	
Healthcare coverage												
Having healthcare coverage^c^	175 (67.0)	70 (47.0)	105 (93.7)	<0.001^***^	1,117 (65.9)	349 (40.8)	768 (91.3)	<0.001^***^	1,292 (66.0)	419 (41.7)	873 (91.6)	<0.001^***^
No healthcare coverage^d^	86 (33.0)	79 (53.0)	7 (6.3)		579 (34.1)	506 (59.2)	73 (8.7)		665 (34.0)	585 (58.3)	80 (8.4)	
Type of healthcare coverage												
UCS	59 (22.6)	-	59 (52.6)	-	389 (22.9)	-	389 (46.2)	-	448 (22.9)	-	448 (47.0)	-
SSS	105 (40.2)	60 (40.3)	45 (40.2)		638 (37.6)	274 (32.0)	364 (43.3)		743 (38.0)	334 (33.3)	409 (42.9)	
CSMBS	1 (0.4)	-	1 (0.9)		15 (0.9)	-	15 (1.8)		16 (0.8)	-	16 (1.7)	
HICS	10 (3.8)	10 (6.7)	-		75 (4.5)	75 (8.8)	-		85 (4.3)	85 (8.5)	-	
Documented without HICS/SSS	76 (29.1)	76 (51.0)	-		479 (28.2)	479 (56.0)	-		555 (28.4)	555 (55.2)	-	
Undocumented without HICS	3 (1.2)	3 (2.0)	-		27 (1.6)	27 (3.2)	-		30 (1.5)	30 (3.0)	-	
OOP payment	7 (2.7)	-	7 (6.3)		73 (4.3)	-	73 (8.7)		80 (4.1)	-	80 (8.4)	
Having medical conditions (*n* = 1,898)^e^												
No condition	124 (49.2)	64 (44.4)	60 (55.6)	0.098^***^	690 (41.9)	328 (40.1)	362 (43.8)	0.134^***^	814 (42.9)	392 (40.7)	422 (45.1)	0.052^***^
Having at least one condition	128 (50.8)	80 (55.6)	48 (44.4)		956 (58.1)	491 (59.9)	465 (56.2)		1,084 (57.1)	571 (59.3)	513 (54.9)	
Disease-specific conditions												
HIV infection (*n* = 1,778)^e^												
No	236 (99.2)	131 (99.2)	105 (99.1)	>0.999^***^	1,525 (99.0)	754 (99.3)	771 (98.7)	0.300^***^	1,761 (99.0)	885 (99.3)	876 (98.8)	0.235^***^
Yes	2 (0.8)	1 (0.8)	1 (0.9)		15 (1.0)	5 (0.7)	10 (1.3)		17 (1.0)	6 (0.7)	11 (1.2)	
Syphilis (*n* = 1,779)^e^												
No	227 (95.8)	129 (98.5)	98 (92.4)	0.046^***^	1,497 (97.1)	750 (98.8)	747 (95.4)	<0.001^***^	1,724 (96.9)	879 (98.8)	845 (95.0)	<0.001^***^
Yes	10 (4.2)	2 (1.5)	8 (7.6)		45 (2.9)	9 (1.2)	36 (4.6)		55 (3.1)	11 (1.2)	44 (5.0)	
Hypertension												
No	256 (98.1)	147 (98.7)	109 (97.3)	0.654^***^	1,646 (97.0)	834 (97.5)	812 (96.5)	0.252^***^	1,902 (97.2)	981 (97.7)	921 (96.6)	0.172^***^
Yes	5 (1.9)	2 (1.3)	3 (2.7)		50 (3.0)	21 (2.5)	29 (3.5)		55 (2.8)	23 (2.3)	32 (3.4)	
Diabetes mellitus												
No	244 (93.5)	139 (93.3)	105 (93.7)	>0.999^***^	1,560 (92.0)	782 (91.5)	778 (92.5)	0.475^***^	1,804 (92.2)	921 (91.7)	883 (92.6)	0.501^***^
Yes	17 (6.5)	10 (6.7)	7 (6.3)		136 (8.0)	73 (8.5)	63 (7.5)		153 (7.8)	83 (8.3)	70 (7.4)	
Obesity (*n* = 1,942)^e^												
No	141 (54.0)	71 (47.7)	70 (62.5)	0.024^***^	805 (47.9)	387 (45.7)	418 (50.1)	0.079^***^	946 (48.7)	458 (46.0)	488 (51.5)	0.016^***^
Yes	120 (46.0)	78 (52.3)	42 (37.5)		876 (52.1)	459 (54.3)	417 (49.9)		996 (51.3)	537 (54.0)	459 (48.5)	
Having pregnancy complications												
No complication	183 (70.1)	108 (72.5)	75 (67.0)	0.343^***^	1,304 (76.9)	672 (78.6)	632 (75.1)	0.095^***^	1,487 (76.0)	780 (77.7)	707 (74.2)	0.072^***^
Having at least one complication	78 (29.9)	41 (27.5)	37 (33.0)		392 (23.1)	183 (21.4)	209 (24.9)		470 (24.0)	224 (22.3)	246 (25.8)	
Specific complications during pregnancy												
Multiple pregnancy												
No	258 (98.8)	147 (98.7)	111 (99.1)	>0.999^***^	1,685 (99.3)	851 (99.5)	834 (99.2)	0.382^***^	1,943 (99.3)	998 (99.4)	945 (99.2)	0.598^***^
Yes	3 (1.2)	2 (1.3)	1 (0.9)		11 (0.7)	4 (0.5)	7 (0.8)		14 (0.7)	6 (0.6)	8 (0.8)	
Anemia (*n* = 1,777)^e^												
No	175 (73.8)	98 (74.8)	77 (72.6)	0.767^***^	1,223 (79.4)	612 (80.5)	611 (78.3)	0.313^***^	1,398 (78.7)	710 (79.7)	688 (77.6)	0.298^***^
Yes	62 (26.2)	33 (25.2)	29 (27.4)		317 (20.6)	148 (19.5)	169 (21.7)		379 (21.3)	181 (20.3)	198 (22.4)	
Chorioamnionitis												
No	261 (100)	149 (100)	112 (100)	-	1,695 (99.9)	855 (100)	840 (99.9)	0.496^***^	1,956 (99.9)	1,004 (100)	952 (99.9)	0.487^***^
Yes	0 (0)	0 (0)	0 (0)		1 (0.1)	0 (0)	1 (0.1)		1 (0.1)	0 (0)	1 (0.1)	
Urinary tract infection												
No	260 (99.6)	149 (100)	111 (99.1)	0.429^***^	1,695 (99.9)	855 (100)	840 (99.9)	0.496^***^	1,955 (99.9)	1,004 (100)	951 (99.8)	0.237^***^
Yes	1 (0.4)	0 (0)	1 (0.9)		1 (0.1)	0 (0)	1 (0.1)		2 (0.1)	0 (0)	2 (0.2)	
Preeclampsia/eclampsia												
No	252 (96.5)	142 (95.3)	110 (98.2)	0.308^***^	1,667 (98.3)	840 (98.2)	827 (98.3)	>0.999^***^	1,919 (98.1)	982 (97.8)	937 (98.3)	0.513^***^
Yes	9 (3.5)	7 (4.7)	2 (1.8)		29 (1.7)	15 (1.8)	14 (1.7)		38 (1.9)	22 (2.2)	16 (1.7)	
PROM												
No	254 (97.3)	146 (98.0)	108 (96.4)	0.467^***^	1,647 (97.1)	835 (97.7)	812 (96.5)	0.193^***^	1,901 (97.1)	981 (97.7)	920 (96.5)	0.136^***^
Yes	7 (2.7)	3 (2.0)	4 (3.6)		49 (2.9)	20 (2.3)	29 (3.5)		56 (2.9)	23 (2.3)	33 (3.5)	
Maternal sepsis												
No	261 (100)	149 (100)	112 (100)	-	1,695 (99.9)	854 (99.9)	841 (100)	>0.999^***^	1,956 (99.9)	1,003 (99.9)	953 (100)	>0.999^***^
Yes	0 (0)	0 (0)	0 (0)		1 (0.1)	1 (0.1)	0 (0)		1 (0.1)	1 (0.1)	0 (0)	
Mode of delivery (*n* = 1,944)^e^												
Normal delivery	160 (61.5)	91 (61.5)	69 (61.6)	0.482^***^	1,065 (63.2)	547 (64.1)	518 (62.4)	0.111^***^	1,225 (63.0)	638 (63.7)	587 (62.3)	0.067^***^
Cesarean delivery	94 (36.2)	52 (35.1)	42 (37.5)		559 (33.2)	269 (31.5)	290 (35.0)		653 (33.6)	321 (32.0)	332 (35.3)	
Vacuum extraction delivery	6 (2.3)	5 (3.4)	1 (0.9)		57 (3.4)	36 (4.2)	21 (2.5)		63 (3.2)	41 (4.1)	22 (2.3)	
Breech delivery	0 (0)	0 (0)	0 (0)		3 (0.2)	2 (0.2)	1 (0.1)		3 (0.2)	2 (0.2)	1 (0.1)	
Receiving COVID-19 vaccination before delivery (*n* = 1,172)^e^												
No	66 (39.8)	36 (42.4)	30 (37.0)	0.528^***^	347 (34.5)	167 (38.8)	180 (31.3)	0.016^***^	413 (35.2)	203 (39.3)	210 (32.0)	0.010^***^
Yes	100 (60.2)	49 (57.6)	51 (63.0)		659 (65.5)	264 (61.2)	395 (68.7)		759 (64.8)	313 (60.7)	446 (68.0)	
1 dose	52 (52.0)	22 (44.9)	30 (58.8)	0.099^***^	229 (34.8)	91 (34.5)	138 (34.9)	0.012^***^	281 (37.0)	113 (36.1)	168 (37.7)	0.003^***^
2 doses	46 (46.0)	27 (55.1)	19 (37.3)		410 (62.2)	171 (64.7)	239 (60.5)		456 (60.1)	198 (63.3)	258 (57.8)	
3 doses	2 (2.0)	0 (0)	2 (3.9)		20 (3.0)	2 (0.8)	18 (4.6)		22 (2.9)	2 (0.6)	20 (4.5)	
**Neonatal characteristics (*n* = 1,971)**												
Sex												
Male	132 (50.0)	74 (49.0)	58 (51.3)	0.804^***^	892 (52.3)	436 (50.8)	456 (53.8)	0.226^***^	1,024 (51.9)	510 (50.5)	514 (53.5)	0.191^***^
Female	132 (50.0)	77 (51.0)	55 (48.7)		815 (47.7)	423 (49.2)	392 (46.2)		947 (48.1)	500 (49.5)	447 (46.5)	
Gestational age at birth, weeks												
Mean ± SD	38.5 ± 1.9	38.6 ± 1.9	38.3 ± 1.7	0.145^**^	38.5 ± 1.8	38.7 ± 1.7	38.3 ± 1.7	<0.001^**^	38.5 ± 1.8	38.7 ± 1.8	38.3 ± 1.7	<0.001^**^
Min – Max	28.0 – 41.6	28.0 – 41.4	30.0 – 41.6		26.0 – 44.1	28.1 – 44.1	26.0 – 41.4		26.0 – 44.1	28.0 – 44.1	26.0 – 41.6	
Term (37 – <42)	238 (90.1)	137 (90.7)	101 (89.4)	0.835^***^	1,542 (90.3)	778 (90.5)	764 (90.1)	0.129^***^	1,780 (90.3)	915 (90.6)	865 (90.0)	0.129^***^
Preterm (<37)	26 (9.9)	14 (9.3)	12 (10.6)		161 (9.4)	77 (9.0)	84 (9.9)		187 (9.5)	91 (9.0)	96 (10.0)	
Post-term (≥42)	0 (0)	0 (0)	0 (0)		4 (0.3)	4 (0.5)	0 (0)		4 (0.2)	4 (0.4)	0 (0)	
Birth weight, grams (*n* = 1,969)^e^												
Mean ± SD	3,107.2 ± 529.9	3,125.0 ± 552.4	3,083.4 ± 499.7	0.528^**^	3,070.7 ± 492.2	3,111.5 ± 492.3	3,029.3 ± 488.9	0.001^**^	3,075.6 ± 497.4	3,113.6 ± 501.5	3,035.7 ± 490.2	0.001^**^
Min – Max	1,090–4,515	1,090–4,515	1,515–4,480		845 – 5,252	1,140–5,252	845 – 4,550		845 – 5,252	1,090–5,252	845 – 4,550	
Normal (2,500–3,999)	227 (86.0)	131 (86.7)	96 (85.0)	0.791^***^	1,495 (87.7)	758 (88.4)	737 (87.0)	0.046^***^	1,722 (87.5)	889 (88.1)	833 (86.8)	0.033^***^
Low (<2,500)	24 (9.1)	12 (8.0)	12 (10.6)		160 (9.4)	69 (8.0)	91 (10.8)		184 (9.3)	81 (8.0)	103 (10.7)	
High (≥4,000)	13 (4.9)	8 (5.3)	5 (4.4)		50 (2.9)	31 (3.6)	19 (2.2)		63 (3.2)	39 (3.9)	24 (2.5)	
Adverse birth outcomes (*n* = 1,971)												
Preterm												
No	238 (90.1)	137 (90.7)	101 (89.4)	0.835^***^	1,546 (90.6)	782 (91.0)	764 (90.1)	0.509^***^	1,784 (90.5)	919 (91.0)	865 (90.0)	0.489^***^
Yes	26 (9.9)	14 (9.3)	12 (10.6)		161 (9.4)	77 (9.0)	84 (9.9)		187 (9.5)	91 (9.0)	96 (10.0)	
Low birth weight (*n* = 1,969)^e^												
No	240 (90.9)	139 (92.0)	101 (89.4)	0.519^***^	1,545 (90.6)	789 (92.0)	756 (89.3)	0.057^***^	1,785 (90.7)	928 (92.0)	857 (89.3)	0.044^***^
Yes	24 (9.1)	12 (8.0)	12 (10.6)		160 (9.4)	69 (8.0)	91 (10.7)		184 (9.3)	81 (8.0)	103 (10.7)	

ANC, antenatal care; COVID-19, coronavirus disease; CSMBS, Civil Servant Medical Benefit Scheme; HICS, Health Insurance Card Scheme; HIV, human immunodeficiency virus; OOP, Out-of-pocket; PROM, premature rupture of membranes; SARS-CoV-2, severe acute respiratory syndrome coronavirus 2; SD, standard deviation; SSS, Social Security Scheme; UCS, Universal Coverage Scheme ^a^ Column percentage; ^b^ Row percentage; ^c^ Healthcare coverage group included UCS, SSS, CSMBS, and HICS; ^d^ No healthcare coverage group included documented migrants without HICS/SSS, undocumented migrants without HICS, and OOP payment (Note: Healthcare coverage for Thai citizen: UCS, SSS, and CSMBS; for immigrants: SSS and HICS); ^e^ Variable with missing data *Independent samples *t*-test with unequal variances; **Independent samples *t*-test; ***Fisher’s exact test

**Table 2:** Univariable analysis of the association between each independent variable and preterm birth outcome

Variables	Preterm birth	No preterm birth	Univariable model^b^		
	*n* (%)^a^	*n* (%)^a^	cRR	95% CI	*P*-value
Total (*n* = 1,971)	187 (9.5)	1,784 (90.5)			
Main independent variable					
Maternal migrant status					
Thai	96 (10.0)	865 (90.0)	1.00	Reference	
Migrant	91 (9.0)	919 (91.0)	0.90	0.69, 1.18	0.458
Covariates					
Maternal SARS-CoV-2 infection during pregnancy					
No	161 (9.4)	1,546 (90.6)	1.00	Reference	
Yes	26 (9.9)	238 (90.1)	1.04	0.70, 1.55	0.830
Maternal age groups (years)					
Normal age (20–34)	136 (9.3)	1,329 (90.7)	1.00	Reference	
Teenage (<20)	23 (12.0)	169 (88.0)	1.29	0.85, 1.96	0.229
Advanced age (≥35)	28 (8.9)	286 (91.1)	0.96	0.65, 1.42	0.839
Healthcare coverage					
Having healthcare coverage	131 (10.1)	1,171 (89.9)	1.00	Reference	
No healthcare coverage	56 (8.4)	613 (91.6)	0.83	0.62, 1.12	0.228
Disease-specific conditions					
HIV					
No	181 (10.2)	1,594 (89.8)	1.00	Reference	
Yes	2 (11.8)	15 (88.2)	1.15	0.31, 4.27	0.831
Syphilis					
No	175 (10.1)	1,562 (89.9)	1.00	Reference	
Yes	7 (12.5)	49 (87.5)	1.24	0.58, 2.64	0.576
Hypertension					
No	184 (9.6)	1,731 (90.4)	1.00	Reference	
Yes	3 (5.4)	53 (94.6)	0.56	0.18, 1.69	0.302
Diabetes mellitus					
No	174 (9.6)	1,643 (90.4)	1.00	Reference	
Yes	13 (8.4)	141 (91.6)	0.88	0.51, 1.51	0.647
Obesity					
No	102 (10.7)	850 (89.3)	1.00	Reference	
Yes	85 (8.5)	919 (91.5)	0.79	0.60, 1.04	0.092
Specific complications during pregnancy					
Multiple pregnancy					
No	177 (9.1)	1,766 (90.9)	1.00	Reference	
Yes	10 (35.7)	18 (64.3)	3.92	2.34, 6.57	<0.001
Anemia					
No	139 (9.9)	1,269 (90.1)	1.00	Reference	
Yes	44 (11.5)	339 (88.5)	1.16	0.85, 1.60	0.353
Chorioamnionitis^c^					
No	187 (9.5)	1,783 (90.5)	-	-	-
Yes	0 (0)	1 (100)	-	-	-
Urinary tract infection^c^					
No	187 (9.5)	1,782 (90.5)	-	-	-
Yes	0 (0)	2 (100)	-	-	-
Preeclampsia/eclampsia					
No	172 (8.9)	1,761 (91.1)	1.00	Reference	
Yes	15 (39.5)	23 (60.5)	4.44	2.92, 6.74	<0.001
PROM					
No	171 (8.9)	1,742 (91.1)	1.00	Reference	
Yes	16 (27.6)	42 (72.4)	3.09	1.99, 4.80	<0.001
Maternal sepsis^c^					
No	187 (9.5)	1,783 (90.5)	-	-	-
Yes	0 (0)	1 (100)	-	-	-
Receiving COVID-19 vaccination before delivery					
Yes	79 (10.3)	685 (89.7)	1.00	Reference	
No	37 (8.9)	379 (91.1)	0.86	0.59, 1.25	0.427

CI, confidence interval; COVID-19, coronavirus disease; cRR, crude risk ratio; HIV, human immunodeficiency virus; PROM, premature rupture of membranes; SARS-CoV-2, severe acute respiratory syndrome coronavirus 2 ^a^ Row percentage ^b^ Univariable analysis was estimated by Poisson regression with robust standard errors. ^c^ Chorioamnionitis, urinary tract infection, and maternal sepsis were not included in univariable model due to no preterm birth outcome.

### Univariable analyses of factors associated with preterm birth

Findings from univariable analyses of independent variables on preterm birth, using Poisson regression with robust standard errors, are presented in Table 2. Regarding the main independent variable of maternal migrant status, being a foreign migrant mother did not increase the risk of preterm birth compared to Thai mothers, as reflected by a crude risk ratio (RR) of 0.9. In contrast, complications during pregnancy, including multiple pregnancy, preeclampsia and eclampsia, and PROM significantly increased the risk of preterm birth as reflected by the magnitude and direction of crude RR of 3.92, 4.44, and 3.09, respectively (Table 2).

### Multivariable analyses of factors associated with preterm birth

Multivariable analyses of the association between maternal migrant status and the risk of preterm birth, adjusting for other covariates, are presented in Table 3. Model 1 served as the unadjusted baseline for comparison with the adjusted RR values obtained from subsequent models. As revealed by subsequent models adjusting for different sets of potential confounding factors (Models 2–8), as depicted in the DAGs in Figure 1, adjusted RR estimates for the association between maternal migrant status and preterm birth ranged from 0.92 to 0.96, all with non-significant confidence interval estimates and *P* values. These estimates consistently indicated that foreign migrant mothers did not have a higher risk of preterm birth compared to Thai mothers (Table 3).

**Table 3:** Multivariable analyses of the association between maternal migrant status and preterm birth outcome, adjusting for other covariates

Variables	Model 1: Unadjusted model		Model 2: Adjusted for maternal age		Model 3: Adjusted for maternal age and obesity		Model 4: Adjusted for maternal age, obesity, and diabetes mellitus	
	RR (95% CI)	*P*-value	RR (95% CI)	*P*-value	RR (95% CI)	*P*-value	RR (95% CI)	*P*-value
Main independent variable								
Maternal migrant status								
Thai	Reference		Reference		Reference		Reference	
Migrant	0.90 (0.69, 1.18)	0.458	0.94 (0.71, 1.25)	0.671	0.94 (0.71, 1.25)	0.690	0.94 (0.71, 1.25)	0.689
Covariates								
Maternal age (years)								
Normal age (20–34)			Reference		Reference		Reference	
Teenage (<20)			1.26 (0.82, 1.93)	0.298	1.19 (0.77, 1.83)	0.435	1.18 (0.77, 1.83)	0.445
Advanced age (≥35)			0.96 (0.65, 1.42)	0.844	0.99 (0.67, 1.46)	0.951	0.99 (0.67, 1.48)	0.975
Obesity								
No					Reference		Reference	
Yes					0.81 (0.61, 1.07)	0.136	0.81 (0.61, 1.07)	0.140
Diabetes mellitus								
No							Reference	
Yes							0.94 (0.54, 1.64)	0.828
Hypertension								
No								
Yes								
Preeclampsia/eclampsia								
No								
Yes								
PROM								
No								
Yes								
Anemia								
No								
Yes								
**Variables**	**Model 5:** **Adjusted for maternal age, obesity, and hypertension**		**Model 6:** **Adjusted for maternal age, obesity, hypertension, and preeclampsia/eclampsia**		**Model 7:** **Adjusted for maternal age, obesity, hypertension, preeclampsia/eclampsia, and PROM**		**Model 8:** **Adjusted for maternal age and anemia**	
	**RR (95% CI)**	***P*-value**	**RR (95% CI)**	***P*-value**	**RR (95% CI)**	***P*-value**	**RR (95% CI)**	***P*-value**
Main independent variable								
Maternal migrant status								
Thai	Reference		Reference		Reference		Reference	
Migrant	0.94 (0.71, 1.24)	0.661	0.92 (0.70, 1.22)	0.571	0.94 (0.71, 1.24)	0.653	0.96 (0.72, 1.27)	0.775
Covariates								
Maternal age (years)								
Normal age (20–34)	Reference		Reference		Reference		Reference	
Teenage (<20)	1.18 (0.77, 1.82)	0.454	1.22 (0.79, 1.87)	0.365	1.16 (0.75, 1.78)	0.503	1.21 (0.79, 1.86)	0.381
Advanced age (≥35)	1.00 (0.67, 1.47)	0.990	1.01 (0.69, 1.50)	0.942	1.02 (0.70, 1.51)	0.905	0.95 (0.64, 1.41)	0.793
Obesity								
No	Reference		Reference		Reference			
Yes	0.82 (0.62, 1.08)	0.165	0.77 (0.58, 1.02)	0.064	0.76 (0.58, 1.01)	0.056		
Diabetes mellitus								
No								
Yes								
Hypertension								
No	Reference		Reference		Reference			
Yes	0.60 (0.20, 1.83)	0.371	0.55 (0.19, 1.60)	0.269	0.56 (0.19, 1.63)	0.287		
Preeclampsia/eclampsia								
No			Reference		Reference			
Yes			4.95 (3.23, 7.60)	<0.001	5.26 (3.42, 8.09)	<0.001		
PROM								
No					Reference			
Yes					3.20 (2.03, 5.03)	<0.001		
Anemia								
No							Reference	
Yes							1.15 (0.83, 1.58)	0.407

CI, confidence interval; PROM, premature rupture of membranes; RR, risk ratio; Multivariable models were estimated by Poisson regression with robust standard errors.

**Table 4:** Univariable analysis of the association between each independent variable and the low birth weight outcome

Variables	Low birth weight	No low birth weight	Univariable model^b^		
	*n* (%)^a^	*n* (%)^a^	cRR	95% CI	*P*-value
Total (*n* = 1,969)	184 (9.3)	1,785 (90.7)			
Main independent variable					
Maternal migrant status					
Thai	103 (10.7)	857 (89.3)	1.00	Reference	
Migrant	81 (8.0)	928 (92.0)	0.75	0.57, 0.99	0.040
Covariates					
Maternal SARS-CoV-2 infection during pregnancy					
No	160 (9.4)	1,545 (90.6)	1.00	Reference	
Yes	24 (9.1)	240 (90.9)	0.97	0.64, 1.46	0.879
Maternal age groups (years)					
Normal age (20–34)	116 (7.9)	1,348 (92.1)	1.00	Reference	
Teenage (<20)	34 (17.7)	158 (82.3)	2.23	1.57, 3.18	<0.001
Advanced age (≥35)	34 (10.9)	279 (89.1)	1.37	0.95, 1.97	0.088
Healthcare coverage					
Having healthcare coverage	127 (9.8)	1,174 (90.2)	1.00	Reference	
No healthcare coverage	57 (8.5)	611 (91.5)	0.87	0.65, 1.18	0.377
Disease-specific conditions					
HIV					
No	179 (10.1)	1,594 (89.9)	1.00	Reference	
Yes	2 (11.8)	15 (88.2)	1.17	0.31, 4.32	0.819
Syphilis					
No	171 (9.9)	1,564 (90.1)	1.00	Reference	
Yes	9 (16.1)	47 (83.9)	1.63	0.83, 3.19	0.153
Hypertension					
No	179 (9.4)	1,734 (90.6)	1.00	Reference	
Yes	5 (8.9)	51 (91.1)	0.95	0.41, 2.23	0.914
Diabetes mellitus					
No	175 (9.6)	1,640 (90.4)	1.00	Reference	
Yes	9 (5.8)	145 (94.2)	0.61	0.32, 1.16	0.131
Obesity					
No	113 (11.9)	838 (88.1)	1.00	Reference	
Yes	69 (6.9)	934 (93.1)	0.58	0.43, 0.77	<0.001
Specific complications during pregnancy					
Multiple pregnancy					
No	166 (8.6)	1,775 (91.4)	1.00	Reference	
Yes	18 (64.3)	10 (35.7)	7.52	5.50, 10.27	<0.001
Anemia					
No	129 (9.2)	1,278 (90.8)	1.00	Reference	
Yes	52 (13.6)	330 (86.4)	1.48	1.10, 2.01	0.010
Chorioamnionitis^c^					
No	184 (9.4)	1,784 (90.6)	-	-	-
Yes	0 (0)	1 (100)	-	-	-
Urinary tract infection^c^					
No	184 (9.4)	1,783 (90.6)	-	-	-
Yes	0 (0)	2 (100)	-	-	-
Preeclampsia/eclampsia					
No	171 (8.9)	1,760 (91.1)	1.00	Reference	
Yes	13 (34.2)	25 (65.8)	3.86	2.43, 6.14	<0.001
PROM					
No	170 (8.9)	1,741 (91.1)	1.00	Reference	
Yes	14 (24.1)	44 (75.9)	2.71	1.68, 4.38	<0.001
Maternal sepsis^c^					
No	184 (9.4)	1,784 (90.6)	-	-	-
Yes	0 (0)	1 (100)	-	-	-
Receiving COVID-19 vaccination before delivery					
Yes	75 (9.8)	688 (90.2)	1.00	Reference	
No	31 (7.5)	384 (92.5)	0.76	0.51, 1.14	0.180

CI, confidence interval; COVID-19, coronavirus disease; cRR, crude risk ratio; HIV, human immunodeficiency virus; PROM, premature rupture of membranes; SARS-CoV-2, severe acute respiratory syndrome coronavirus 2 ^a^ Row percentage ^b^ Univariable analysis was estimated by Poisson regression with robust standard error. ^c^ Chorioamnionitis, urinary tract infection, and maternal sepsis were not included in univariable model due to no low-birth-weight outcome.

### Univariable analyses of factors associated with low birth weight

Results from univariable analyses of independent variables on the outcome of low birth weight are presented in Table 4. Being foreign migrant mothers was significantly associated with a lower risk of low birth weight compared to Thai mothers, as shown by a crude RR of 0.75 (*P* = 0.040). Other covariates that were significantly associated with a higher risk of low birth weight in newborns included teenage mothers aged under 20 (crude RR = 2.23), multiple pregnancy (crude RR = 7.52), pregnancy anemia (crude RR = 1.48), pre-eclampsia and eclampsia (crude RR = 3.86), and PROM (crude RR = 2.71). In contrast, obese mothers were significantly associated with a lower risk of low birth weight (crude RR = 0.58; Table 4).

### Multivariable analyses of factors associated with low birth weight

Findings from multivariable analyses of the association between maternal migrant status and the risk of low birth weight, adjusting for other covariates, are presented in Table 5. Model 1, an unadjusted model, indicated that foreign migrant mothers had a significantly lower risk of low birth weight compared to Thai mothers (crude RR = 0.75; *P* = 0.040). Subsequent models for the association between maternal migrant status and low birth weight, adjusting for varying sets of covariates according to the DAGs in Figure 2 (Models 2–8), showed adjusted RR estimates ranging from 0.84 to 0.86, with non-significant confidence intervals and *P* values. The findings consistently indicated that foreign migrant mothers did not have a significantly lower risk of low birth weight compared to Thai mothers (Table 5).

**Table 5:** Multivariable analyses of the association between maternal migrant status and low birth weight outcome, adjusting for other covariates

Variables	Model 1: Unadjusted model		Model 2: Adjusted for maternal age		Model 3: Adjusted for maternal age and obesity		Model 4: Adjusted for maternal age, obesity, and diabetes mellitus	
	RR (95% CI)	*P*-value	RR (95% CI)	*P*-value	RR (95% CI)	*P*-value	RR (95% CI)	*P*-value
Main independent variable								
Maternal migrant status								
Thai	Reference		Reference		Reference		Reference	
Migrant	0.75 (0.57, 0.99)	0.040	0.86 (0.64, 1.15)	0.309	0.86 (0.64, 1.15)	0.303	0.86 (0.64, 1.15)	0.300
Covariates								
Maternal age (years)								
Normal age (20–34)			Reference		Reference		Reference	
Teenage (<20)			2.09 (1.45, 3.02)	<0.001	1.81 (1.24, 2.64)	0.002	1.78 (1.22, 2.60)	0.003
Advanced age (≥35)			1.38 (0.96, 1.98)	0.085	1.47 (1.02, 2.10)	0.038	1.51 (1.05, 2.18)	0.026
Obesity								
No					Reference		Reference	
Yes					0.61 (0.46, 0.82)	0.001	0.62 (0.46, 0.84)	0.002
Diabetes mellitus								
No							Reference	
Yes							0.68 (0.35, 1.32)	0.251
Hypertension								
No								
Yes								
Preeclampsia/eclampsia								
No								
Yes								
PROM								
No								
Yes								
Anemia								
No								
Yes								
**Variables**	**Model 5:** **Adjusted for maternal age, obesity, and hypertension**		**Model 6:** **Adjusted for maternal age, obesity, hypertension, and preeclampsia/eclampsia**		**Model 7:** **Adjusted for maternal age, obesity, hypertension, preeclampsia/eclampsia, and PROM**		**Model 8:** **Adjusted for maternal age and anemia**	
	**RR (95% CI)**	***P*-value**	**RR (95% CI)**	***P*-value**	**RR (95% CI)**	***P*-value**	**RR (95% CI)**	***P*-value**
Main independent variable								
Maternal migrant status								
Thai	Reference		Reference		Reference		Reference	
Migrant	0.86 (0.64, 1.15)	0.307	0.84 (0.63, 1.12)	0.240	0.85 (0.64, 1.14)	0.272	0.86 (0.64, 1.15)	0.298
Covariates								
Maternal age (years)								
Normal age (20–34)	Reference		Reference		Reference		Reference	
Teenage (<20)	1.81 (1.24, 2.64)	0.002	1.87 (1.28, 2.72)	0.001	1.79 (1.22, 2.63)	0.003	1.96 (1.35, 2.83)	<0.001
Advanced age (≥35)	1.46 (1.02, 2.10)	0.041	1.48 (1.04, 2.13)	0.032	1.50 (1.05, 2.14)	0.027	1.43 (0.99, 2.05)	0.053
Obesity								
No	Reference		Reference		Reference			
Yes	0.61 (0.45, 0.82)	0.001	0.57 (0.42, 0.77)	<0.001	0.56 (0.42, 0.76)	<0.001		
Diabetes mellitus								
No								
Yes								
Hypertension								
No	Reference		Reference		Reference			
Yes	1.16 (0.49, 2.73)	0.733	1.06 (0.43, 2.59)	0.896	1.08 (0.44, 2.65)	0.859		
Preeclampsia/eclampsia								
No			Reference		Reference			
Yes			4.95 (3.09, 7.92)	<0.001	5.18 (3.23, 8.30)	<0.001		
PROM								
No					Reference			
Yes					2.69 (1.63, 4.42)	<0.001		
Anemia								
No							Reference	
Yes							1.43 (1.05, 1.94)	0.022

CI, confidence interval; PROM, premature rupture of membranes; RR, risk ratio; Multivariable models were estimated by Poisson regression with robust standard errors.

## DISCUSSION

The incidence of preterm birth was not significantly different between migrant and Thai women. However, the incidence of low birth weight was significantly lower among migrant women compared to Thai women. Univariable and multivariable analyses suggested that being a foreign migrant did not increase the risk of preterm birth compared to Thai mothers. Multivariable analyses indicated that newborns of foreign migrant mothers were not significantly associated with a lower risk of low birth weight compared to those of Thai mothers. Finally, the overall incidence of SARS-CoV-2 infection was 13.3% at the peak of the epidemic, with a significantly higher risk of infection during pregnancy observed among foreign migrant mothers.

A plausible explanation for the absence of significant differences in the risk of preterm birth and low birth weight between the newborns of migrant and Thai mothers in this context may be attributed to selective migration, wherein healthier individuals are more likely to migrate for employment, especially in labor-intensive settings. This is particularly relevant to this province, a significant hub of Thailand’s fisheries industry that attracts a substantial foreign migrant workforce. This suggests that self-selection among migrants may influence health outcomes and should be taken into account when comparing migrant and native populations. Furthermore, unlike the settings where migrant women encounter barriers to accessing antenatal care [2], the provision of access to such services in this study context, as part of a humanitarian support initiative, irrespective of documentation status, together with the availability of interpreters to facilitate communication with healthcare providers, may have improved prenatal care accessibility and contributed to reducing disparities in birth outcomes. This may imply the importance of accessible and culturally competent care in reducing health disparities. In addition, as noted in the Supplementary File 1: ‘*For both Thais and migrants with OOP payments who are unable to pay, the hospital’s social welfare or installment payment system will be the last support’* thereby ensuring humanitarian assistance aimed at reducing disparities in insurance coverage and financial barriers to maternal and child health services.

Maternal age was considered a potential confounder in Model 2 (E ← A → D), as it may affect the risk of preterm birth and the mother’s migrant status (Figure 1). GA at birth was significantly higher for foreign migrant mothers compared to Thai mothers. The high incidence of teenage pregnancies among Thai mothers, which is associated with a greater risk of preterm birth [21], may explain this difference. Immigrants who enter the country must be at least 15 years old to perform normal work and 18 years old for hazardous work due to child labor laws [22]. Given that migrants from neighboring countries often choose to come to Thailand for its strong economy and job opportunities [23], this effectively limits the number of migrant teenage mothers entering the country. Furthermore, because foreign migrants are required to undergo pregnancy testing for documentation purposes and may face the risk of unemployment if found to be pregnant, this policy may influence their decision to conceive while residing in Thailand [24].

Maternal obesity was identified as a potential factor influencing the risk of low birth weight in Model 3 (E ← A → O → D) as it may impair placental function and impose fetal growth restriction (Figure 2). Additionally, health conditions associated with obesity, such as chronic inflammation and insulin resistance, may disrupt nutrient transport in the placenta [25]. Despite this, average birth weight was significantly higher among migrant mothers compared to Thai mothers. It appeared that foreign migrant mothers had a higher incidence of obesity compared to Thai mothers. Maternal obesity can be associated with increased birth weight, and infants born to obese mothers generally have higher average birth weights compared to those of mothers with normal weight [26]. This may be attributable to limited knowledge of health and diet education and consumption of calorie-dense foods, such as sweetened beverages and high-sugar, high-fat snacks, leading to excessive gestational weight gain [27]. This may also be attributable to cultural differences and changes in life context, where the shift from food insecurity to an ample food supply encourages consumption.

Although healthcare coverage was not controlled for in the DAG, it was identified as a potential factor influencing preterm birth and low birth weight, as it affects access to vaccination, treatment of existing conditions, prenatal care, and delivery services. Foreign migrant mothers were less likely to have adequate health insurance compared with Thai mothers. This disparity reflects Thailand’s healthcare system, in which Thai citizens are covered under the Universal Coverage Scheme at no cost, whereas foreign migrants must purchase insurance through the Social Security Scheme or the Health Insurance Card Scheme [28]. Despite these differences, no significant association was observed between insurance status and the incidence of preterm birth or low birth weight (Tables 2 and 4). The presence of pre-existing diseases or infections, such as SARS-CoV-2, syphilis, and preeclampsia, was identified as an intermediate factor. Regarding SARS-CoV-2 infection, migrant mothers demonstrated a higher overall incidence compared with Thai mothers. This may be partly explained by the large migrant population residing in the region served by the study hospital. Additionally, the lower socioeconomic conditions commonly experienced by migrants may contribute to a higher risk of communicable diseases [29]. This observation aligns with previous studies reporting increased rates of COVID-19 among migrant communities [16, 17, 23].

Syphilis, another communicable disease of interest, was observed at a higher incidence among Thai mothers. The age difference between Thai and migrant mothers may have contributed to this finding, as younger individuals are less likely to engage in safe sexual practices, increasing the risk of sexually transmitted infections [30]. Further stratification revealed substantial overlap between cases of teenage pregnancy and syphilis among Thai mothers.

This study contributes to the existing literature on birth outcomes among Southeast Asian migrant and Thai mothers during the peak of the COVID-19 pandemic, a period characterized by heightened health disparities, particularly among migrant populations. By employing DAGs, this study enhanced causal inference and understanding of how maternal migrant status contributes to disparities in birth outcomes, with comprehensive analyses of the roles of other social and clinical determinants that may influence the study outcomes. Nonetheless, despite the use of DAGs and efforts to control potential confounders, other unmeasured factors commonly found among foreign migrant mothers, such as language barriers, socioeconomic instability, social support, and occupational conditions, may still influence birth outcomes in this context. Future research on disparities in birth outcomes among newborns of migrant mothers should incorporate these variables to enhance understanding and inform targeted interventions.

This data offers valuable insights into the relationship between migrant status and adverse birth outcomes; however, its generalizability to non-mainland Southeast Asian immigrants may be limited due to the specific population composition studied. The participants were primarily from neighboring Southeast Asian countries and employed in local agricultural and fishery sectors, potentially overlooking other occupational contexts. These characteristics may not reflect the experiences of migrants in other settings, such as urban or service-based environments, or those from non-Southeast Asian backgrounds.

Future studies could examine additional maternal factors, such as socioeconomic status, nutrition, smoking, alcohol use, and parity, to better understand their potential role as confounders in the association between maternal migrant status and preterm birth or low birth weight outcomes. Furthermore, apart from preterm birth and low birth weight, other significant birth outcomes, such as respiratory distress syndrome, congenital pneumonia, and neonatal sepsis, may reveal disparities between migrants and non-migrants. Further investigation into these additional outcomes and other migrant populations is recommended to fully assess the possibility of health inequity among migrants in different contexts.

## CONCLUSION

Maternal migrant status did not significantly affect preterm birth and low birth weight among newborns in this setting. Contrary to the assumption that migrants may face hardships, especially during the COVID-19 pandemic, leading to poor health outcomes, the incidence of preterm birth and low birth weight among the newborns of migrant mothers was not higher compared to that of local mothers in this study. This suggested an immigrant health paradox observed during the COVID-19 pandemic. Although not reflective of typical conditions, this provided valuable insights into migrant health during emergencies. The study’s application of causal inference models and rigorous epidemiological study design was illustrated in maternal and child health research in migrants, suggesting that similar strategies could help mitigate bias in future investigations in this field.

## Supplementary Material



## Data Availability

Data are not available to the public due to hospital restrictions on sharing patients’ clinical data.
