# Supplementary material for: Birth outcome disparities and immigrant paradox among Southeast Asian migrant and Thai mothers during the COVID-19 pandemic: a retrospective cohort study
Source: J Med Life. 2025 Dec;18(12):1108–26. doi: 10.25122/jml-2025-0128 (PMC12871437; doi:10.25122/jml-2025-0128)
Supplement: Supplementary file 1 [file JMedLife-18-1108-s001.pdf]

For maternal health services in Thailand, Thai citizens are covered by one of three public healthcare coverages provided by the Thai government, including:

1. **Social Security Scheme (SSS)** is managed by the Ministry of Labor. In the formal private sector, private employees are registered with SSS (19% of the total population) and must pay into the system every month. They are eligible for free medical services at a single hospital that they have selected and registered with from a list of public and private hospitals.
2. **Civil Servant Medical Benefit Scheme (CSMBS)** is administered by the Ministry of Finance. CSMBS (6% of the total Thai population) automatically covers Thai civil servants and their family members, including parents, spouses, and dependents under 20 years old. This insurance can cover all public hospitals and some university hospitals.
3. **Universal Coverage Scheme (UCS)** is organized by the National Health Security Office. Thai citizens with Thai national identification numbers who are not covered by SSS or CSMBS are covered by UCS, which accounts for around 75% of the total population. The Thais covered by this insurance policy are required to register and be covered by a designated public hospital.

Nevertheless, if Thai people with UCS or SSS receive medical services in other hospitals apart from their registered hospital, they have to pay for those services (out-of-pocket (OOP) payment). Moreover, if Thais are unemployed from the formal private sector, resulting in the end of SSS, and are in the process of UCS registration, they have no healthcare coverage and must pay OOP for services until registered.

For migrants who work in Thailand, there are two types of health coverage provided by the Thai government:

1. SSS. Legal migrants who work as private employees in the formal private sector can register for SSS as Thai citizens.
2. Health Insurance Card Scheme (HICS) is a national insurance scheme for Cambodia, Lao PDR, Myanmar, and Vietnam (CLMV) migrants managed by the Ministry of Public Health. The migrants who are private employees but are not entitled to the SSS due to 1) illegal or undocumented migrant workers, 2) legal migrants working in the informal sectors, or 3) dependents of migrant workers or their children can individually buy the card for HICS annually. However, like the UCS, the migrants have limited coverage by HICS in a specific registered hospital.

In contrast, migrants who have no SSS or HICS have to pay for medical services. This group can be classified as 1) legal migrants (documented migrants without HICS) and 2) illegal migrants (undocumented migrants).

For both Thais and migrants with OOP payments who are unable to pay, the hospital's social welfare or installment payment system will be the last support.

In summary, patients with SSS, CSMBS, UCS, or HICS are insured for healthcare services, while the remainder must pay OOP.

REFERENCES

1. Suphanchaimat R, Putthasri W, Prakongsai P, Tangcharoensathien V. Evolution and complexity of government policies to protect the health of undocumented/illegal migrants in Thailand - the unsolved challenges. Risk Manag Healthc Policy. 2017;10:49-62. doi:10.2147/RMHP.S130442
2. Tangcharoensathien V, Patcharanarumol W, Greetong T, Suwanwela W, Kesthom N, Viriyathorn S, *et al.* Case study: Thailand Universal Coverage Scheme. In: Barber SL, Lorenzoni L, Ong P. Price setting and price regulation in health care: lessons for advancing universal health coverage. World Health Organization; 2019:219-254. Accessed from: https://iris.who.int/bitstream/handle/10665/325547/9789241515924-eng.pdf
3. Tangcharoensathien V, Supachutikul A, Lertiendumrong J. The social security scheme in Thailand: what lessons can be drawn?. Soc Sci Med. 1999;48(7):913-923. doi:10.1016/s0277-9536(98)00392-x

© 2025 by the authors. This article is an open access article distributed under the terms and conditions of the Creative Commons Attribution (CC BY 4.0) license.
